# Testosterone nadir and clinical outcomes in patients with advanced prostate cancer: Post hoc analysis of triptorelin pamoate Phase III studies

**DOI:** 10.1002/bco2.318

**Published:** 2024-01-10

**Authors:** Laurence Klotz, Tri Tat

**Affiliations:** ^1^ Division of Urology, Sunnybrook Health Sciences Centre University of Toronto Toronto Ontario Canada; ^2^ Debiopharm International Lausanne Switzerland

**Keywords:** advanced prostate cancer, clinical trial data, survival, testosterone suppression, triptorelin pamoate

## Abstract

**Objective:**

The objective of the study is to evaluate whether low nadir testosterone during treatment with triptorelin pamoate, a luteinising hormone‐releasing hormone (LHRH) agonist, is associated with improved clinical outcomes in patients with advanced prostate cancer using a retrospective analysis of clinical trial data.

**Patients and methods:**

Data were pooled from three prospective, 9–12‐month Phase III studies of triptorelin monotherapy in patients with advanced prostate cancer (including NCT00751790). The serum testosterone concentration suppression targets evaluated were <0.35 nmol/L (<10 ng/dl), <0.7 nmol/L (<20 ng/dl), <1.7 nmol/L (<50 ng/dl) and ≥1.7 nmol/L. Overall survival (OS) and disease‐specific survival (DSS) by testosterone suppression group were assessed by Kaplan–Meier analysis, with log‐rank test. The time frame for the primary analysis was Days 1–518 (median OS follow‐up 254 days [range, 29–518 days]) and for the sensitivity analyses was Days 1–262. Supplementary analyses combined the ≥0.7‐ to <1.7‐nmol/L and ≥1.7‐nmol/L groups.

**Results:**

The sample size comprised 592 patients (most received triptorelin monotherapy; four reported concomitant androgen receptor‐axis–targeted therapy). Nadir testosterones of <0.35, ≥0.35 to <0.7, ≥0.7 to <1.7 and ≥1.7 nmol/L were achieved by 96%, 3.2%, 0.34% and 0.17% of patients, respectively. Better OS with decreasing level of nadir testosterone was observed (*p* < 0.001) and this persisted after sensitivity/supplemental analyses (all *p* < 0.001). Differences in DSS with decreasing levels of nadir testosterone were not statistically significant in the primary analysis. Sensitivity/supplemental analysis showed better DSS with decreasing level of nadir testosterone (Days 1–262, *p* = 0.01; combined groups Days 1–518, *p* = 0.03; combined groups Days 1–262, *p* = 0.005).

**Conclusion:**

Low nadir testosterone achieved during treatment with the LHRH agonist triptorelin was associated with improved OS and DSS in patients with advanced prostate cancer.

## 1 INTRODUCTION

Lower levels of testosterone in men on androgen‐deprivation therapy have been linked with improved clinical outcomes in advanced prostate cancer.^1^ In the setting of testosterone‐lowering treatment, castration was originally defined as a serum testosterone level <1.7 nmol/L (<50 ng/dl). This target level was widely adopted by regulatory authorities.^2^ Clinical experience utilising contemporary assay technology has suggested that a lower serum testosterone level threshold may be more appropriate than the historic definition.^2^ In the PR‐7 study, nadir testosterone levels ≤0.7 nmol/L (≤20 ng/dl) within the first year of androgen‐deprivation therapy correlated with improved cause‐specific survival and duration of response in men with recurrent, nonmetastatic prostate cancer.^1^

Triptorelin pamoate is a gonadotropin‐releasing hormone agonist indicated for the treatment of advanced prostate cancer.^3^ Pooled data from nine prospective studies showed that 80%–90% of patients with advanced prostate cancer reached testosterone levels <0.7 nmol/L with triptorelin.^4^ Mean testosterone levels were >0.35 nmol/L (>10 ng/dl) at Month 1, and <0.35 nmol/L for Months 2–12.^4^ Testosterone levels continued to decrease between Months 1 and 2.^4^ Serum testosterone levels at the end of the study were lowest with the 1‐month formulation (median, 0.1 nmol/L [3 ng/dl]; interquartile range, 0.1–0.23 nmol/L [3–7 ng/dl]) compared with the 3‐month (0.2; 0.1–0.3 nmol/L [6; 3–9 ng/dl]) and 6‐month formulations (0.3; 0.2–0.5 nmol/L [9; 6–14 ng/dl]).^4^ In a Japanese study of androgen‐deprivation therapy, very low levels of serum testosterone (0.07–0.14 nmol/L [2–4 ng/dl]) were associated with a better prognosis in patients with metastatic prostate cancer and certain *SRD5A2* polymorphisms.^5^

This retrospective study evaluated data from phase III trials of triptorelin conducted by Debiopharm to assess whether nadir testosterone during treatment is a determinant of clinical endpoints in patients with advanced prostate cancer treated with triptorelin.

## 2 PATIENTS AND METHODS

This was a retrospective pooled analysis of data from three prospective, Phase III studies of triptorelin in patients with advanced prostate cancer: DEB‐96‐TRI‐01 1st phase, DEB‐96‐TRI‐01 2nd phase and DEB‐TRI6M‐301 (clinicaltrials.gov identifier: NCT00751790) (Table 1).^4^, ^6^ DEB‐96‐TRI‐01 was not registered in a centralised database because, at the time it was implemented, this was not a requirement. All three studies were conducted in South Africa. The primary objective of the analysis was to explore whether low nadir serum testosterone during androgen‐deprivation treatment improves overall survival (OS) and disease‐specific survival (DSS) due to prostate cancer or complications of cancer treatment. Secondary objectives included exploring the proportion of time that testosterone was suppressed below the target level, the time to achieve a testosterone level below the suppression target and defining prostate‐specific antigen (PSA) kinetics.

**TABLE 1:** Overview of included studies.

Study ID; location	Study design; number of patients	Study population	Treatment formulation evaluated	Treatment period
DEB‐96‐TRI‐01 first phase; South Africa	Phase III, 19 centres, open‐label, randomised, controlled, 2‐arm; *N* = 335	Advanced prostate cancer	Triptorelin pamoate 3.75 mg IM every 4 weeks Triptorelin 11.25 mg IM every 12 weeks	9 months (252 days)
DEB‐96‐TRI‐01 second phase; South Africa	Phase III, 29 centres, open‐label, randomised, controlled, 2‐arm; *N* = 277	Advanced prostate cancer	Triptorelin pamoate 3.75 mg IM every 4 weeks	9 months (252 days)
DEB‐TRI6M‐301; South Africa NCT00751790	Phase III, 13 centres, open‐label, 1‐arm; *N* = 120	Advanced prostate cancer	Triptorelin pamoate 22.5 mg IM every 24 weeks	12 months (336 days)

Abbreviation: IM, intramuscular.

### 2.1 Testosterone measurements and targets

Testosterone‐derived variables were based on testosterone data collected at baseline, during the treatment period and during the 28‐day safety follow‐up period. The serum testosterone concentration suppression targets assessed were defined as <0.35 nmol/L, <0.7 nmol/L and <1.7 nmol/L, based on the historical suppression target level of 1.7 nmol/L ^7^ and currently recommended levels of 0.5–0.7 nmol/L (14–20 ng/dl).^7^ Testosterone was measured by validated radioimmunoassay or liquid chromatography tandem mass spectrometry.^4^ The testosterone lower limit of quantification (LLOQ) was 0.2 nmol/L (6 ng/dl) in studies DEB‐96‐TRI‐01 first phase and DEB‐96‐TRI‐01 second phase, and 0.104 nmol/L (3 ng/dl) in study DEB‐TRI6M‐301.

### 2.2 PSA measurements

In DEB‐TRI6M‐301, blood samples for PSA assessments were taken at 0 h (prior to injection) on Day 1 and again on Days 85, 169 (prior to injection), 253 and 337. In DEB‐96‐TRI‐01, blood samples were obtained on Days 1, 85, 169 and 253 only. Serum PSA (total PSA) levels were measured via automated immunoassay in the local central laboratory (Quintiles Laboratories) in the Republic of South Africa. The LLOQ for PSA was 0.2 μg/L in DEB‐96‐TRI‐01 first phase, 0.2 μg/L in DEB‐96‐TRI‐01 second phase and 0.1 μg/L in DEB‐TRI6M‐301.

### 2.3 Endpoints

The primary efficacy endpoints were OS and DSS. OS was defined as the elapsed time between the first dose and death from any cause. Patients who did not die were censored at the last date of contact. DSS was defined as the elapsed time between first dose and death due to prostate cancer (preferred term of adverse event with fatal outcome: asthenia, malignant neoplasm progression, metastases to liver, prostate cancer and pulmonary mass) or a complication of cancer treatment. Patients who died from other causes or who did not die from prostate cancer or a complication of cancer treatment were censored at the last date of contact.

The primary explanatory risk variable was serum nadir testosterone level achieved during androgen‐deprivation treatment with triptorelin. Secondary explanatory risk variables included the proportion of time with serum testosterone below the suppression target and time to (first) testosterone below the suppression target. Descriptive variables were serum testosterone and PSA concentrations and the change in these variables from baseline.

### 2.4 Statistical analysis

Geometric mean and fold change from baseline in testosterone and PSA were analysed over time. OS and DSS by testosterone suppression group were assessed by Kaplan–Meier (KM) analysis, with log‐rank test. The primary analysis included data from Days 1 to 518 (median OS follow‐up, 254 days [range, 29–518 days]) and the sensitivity analysis from Days 1 to 262, during which time the KM estimate was stable. Patients were categorised into testosterone suppression groups based on nadir testosterone of <0.35 nmol/L, ≥0.35 to <0.7 nmol/L, ≥0.7 to <1.7 nmol/L and ≥1.7 nmol/L. A landmark analysis was also carried out using Day 57 as the landmark date for assigning the testosterone suppression groups. Day 57 was selected as the landmark date as it enabled classification of 99% patients attaining <1.7 nmol/L, 95% attaining <0.7 nmol/L and 80% attaining <0.35 nmol/L suppression by Day 57.

## 3 RESULTS

The intention‐to‐treat (ITT) analysis set included a total of 592 patients (Table 2). Across the studies, most patients (54%) were White (range, 47%–64%) with a mean age of 70.6 years (range, 69.7–71.1 years) [Correction added on 12 February 2024, after first online publication: A percent sign has been added after 47 in the preceding sentence]. The median duration of disease prior to treatment initiation was 1.1 months (range, 0.6–13.4 months). Most patients were diagnosed with advanced T‐stage disease (91% with T3–4 disease; range, 86%–96%). Most patients could not be assessed for regional lymph nodes (83% were NX; range, 81%–87%) and variable rates of metastatic disease were observed (31% were M0, range, 13%–41%; 34% were M1, range, 8%–44%; 35% were MX, range, 22%–79%). Staging was established by histology or cytology for all patients. Four patients reported concomitant use of androgen receptor‐axis–targeted therapy.

**TABLE 2:** Summary of demographic and baseline disease characteristics (ITT).

	DEB‐96‐TRI‐01 first phase	DEB‐96‐TRI‐01 second phase	DEB‐96‐TRI‐01 combined first + second phase	DEB‐96‐TRI‐01 1st phase	DEB‐TRI6M‐301	All studies
Statistic	Triptorelin 3.75 mg (1‐month) ITT *N* = 164	Triptorelin 3.75 mg (1‐month) ITT *N* = 137	Triptorelin 3.75 mg (1‐month) ITT *N* = 301	Triptorelin 11.25 mg (3‐month) ITT *N* = 171	Triptorelin 22.5 mg (6‐month) ITT *N* = 120	Triptorelin all doses ITT *N* = 592
Race, *n* (%)						
*N*	164	137	301	171	120	592
White	81 (49)	80 (58)	161 (53)	80 (47)	77 (64)	318 (54)
Black	59 (36)	37 (27)	96 (32)	64 (37)	27 (23)	187 (32)
Mixed race	24 (15)	19 (14)	43 (14)	27 (16)	16 (13)	86 (15)
Other	0	1 (0.73)	1 (0.33)	0	0	1 (0.17)
Age (years)						
*N*	164	131	295	171	120	586
Mean (*SD*)	70.9 (7.8)	70.8 (8.9)	70.8 (8.3)	69.7 (8.8)	71.1 (8.5)	70.6 (8.5)
Height (cm)						
*N*	163	136	299	170	120	589
Mean (*SD*)	173.0 (8.3)	172.8 (8.7)	172.9 (8.5)	171.1 (8.2)	173.4 (8.2)	172.5 (8.4)
Weight (kg)						
*N*	164	136	300	170	119	589
Mean (*SD*)	73.2 (16.6)	76.2 (15.5)	74.5 (16.2)	72.8 (17.4)	83.5 (16.4)	75.8 (17.0)
Body mass index (kg/m^2^)						
*N*	163	135	298	170	119	587
Mean (*SD*)	24.3 (5.0)	25.5 (4.4)	24.9 (4.7)	24.7 (4.9)	27.7 (4.4)	25.4 (4.8)
Age at diagnosis (years)						
*N*	154	131	285	164	119	568
Mean (*SD*)	70.1 (7.8)	69.6 (8.8)	69.9 (8.3)	69.0 (8.8)	67.8 (8.5)	69.2 (8.5)
Median	70.0	71.0	70.0	68.5	68.0	70
Duration of disease (months)						
*N*	159	137	296	170	119	585
Mean (*SD*)	6.7 (20.0)	8.2 (21.6)	7.4 (20.7)	6.7 (20.1)	34.1 (43.2)	12.6 (28.8)
Median	0.8	0.7	0.7	0.6	13.4	1.1
Stage T, *n* (%)						
*N*	164	137	301	171	120	592
0	0	2 (1.5)	2 (0.66)	0	0	2 (0.34)
1	1 (0.61)	0	1 (0.33)	3 (1.8)	3 (2.5)	7 (1.2)
2	6 (3.7)	1 (0.73)	7 (2.3)	4 (2.3)	11 (9.2)	22 (3.7)
3	98 (60)	83 (61)	181 (60)	108 (63)	72 (60)	361 (61)
4	52 (32)	49 (36)	101 (34)	46 (27)	31 (26)	178 (30)
X	2 (1.2)			2 (1.5)	4 (1.3)	12 (2.0)
Other	5 (3.0)	0	5 (1.7)	5 (2.9)	0	10 (1.7)
Stage N, *n* (%)						
*N*	164	137	301	171	120	592
0	27 (16)	10 (7.3)	37 (12)	25 (15)	15 (13)	77 (13)
1	4 (2.4)	6 (4.4)	10 (3.3)	8 (4.7)	1 (0.83)	19 (3.2)
2	1 (0.61)	1 (0.73)	2 (0.66)	0	0	2 (0.34)
3	0	1 (0.73)	1 (0.33)	0	0	1 (0.17)
X	132 (80)	119 (87)	251 (83)	138 (81)	104 (87)	493 (83)
Stage M, *n* (%)						
*N*	164	137	301	171	120	592
0	56 (34)	56 (41)	112 (37)	59 (35)	15 (13)	186 (31)
1	70 (43)	47 (34)	117 (39)	75 (44)	10 (8.3)	202 (34)
X	38 (23)	34 (25)	72 (24)	37 (22)	95 (79)	204 (34)
Androgen receptor‐axis targeted therapy, *n* (%)						
*N*	164	137	301	171	120	592
Prior medication	0	0	0	0	0	0
Concomitant medication	0	2 (1.5)	2 (0.66)	0	2 (1.7)	4 (0.68)

*Note*: All patients were male. All patients had staging established by histology/cytology. DEB‐96‐TRI‐01 1st phase and DEB‐96‐TRI‐01 2nd phase TNM staging according to Schroder et al.^8^ ‘DEB‐TRI6M‐301 TNM staging according to International Union Against Cancer (UICC) TNM classification of malignant tumours, 6th edition, 2002.^9^

Abbreviations: ITT, intention to treat; *SD*, standard deviation; X, cannot be assessed.

### 3.1 Testosterone concentrations

**FIGURE 1:**
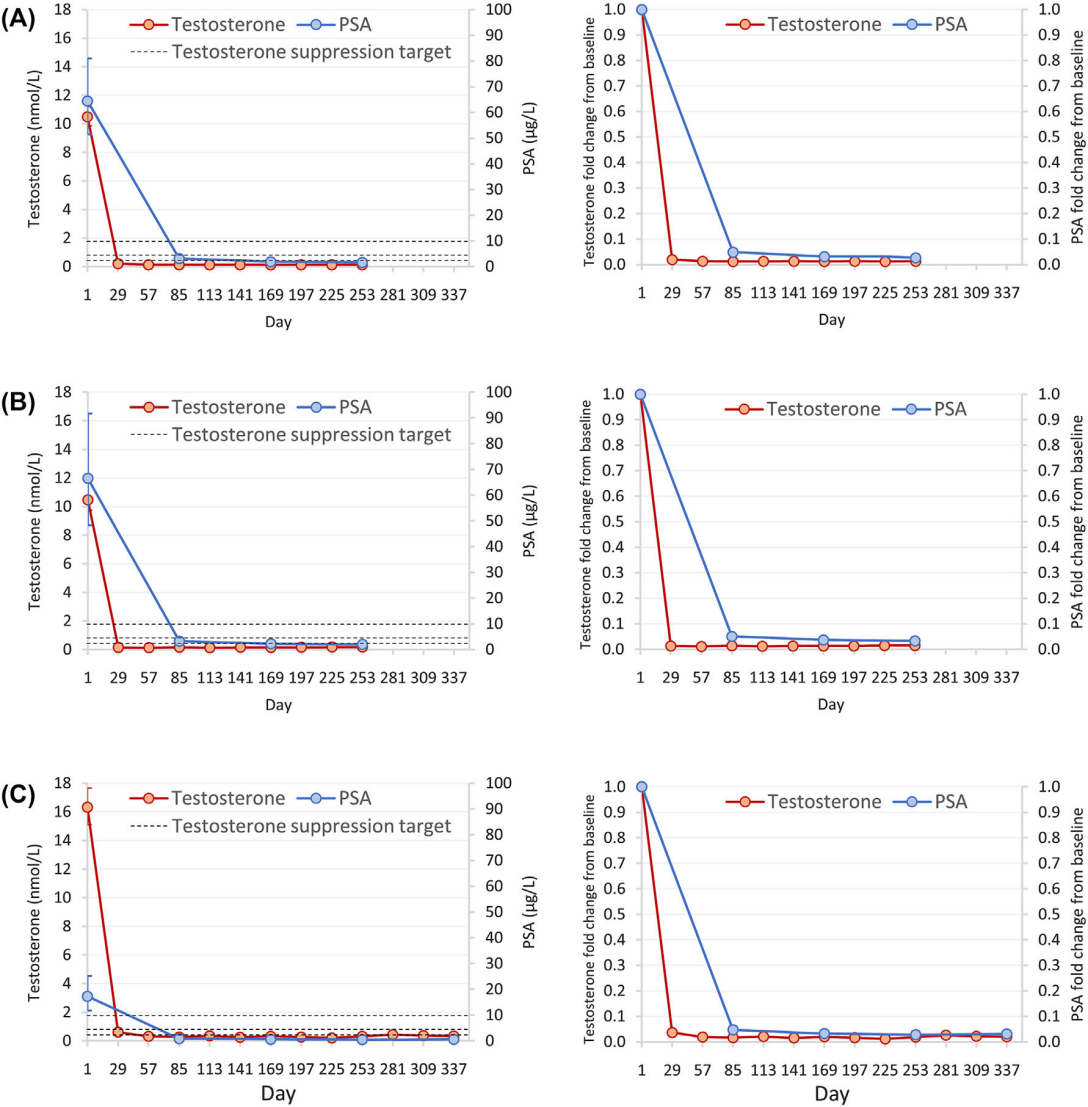
Geometric mean (left panels) and fold change from baseline (right panels) over time in testosterone and PSA concentrations in patients treated with (A) triptorelin 3.75 mg (1‐month formulation), (B) triptorelin 11.25 mg (3‐month formulation) and (C) triptorelin 22.5 mg (6‐month formulation) (ITT analysis set). Dotted reference lines in left panels correspond to testosterone suppression targets: <0.35 nmol/L (<10 ng/dl), <0.7 nmol/L (<20 ng/dl), <1.7 nmol/L (<50 ng/dl). To convert testosterone concentrations from nmol/L to ng/dL, divide by 0.0347. ITT, intention to treat; PSA, prostate‐specific antigen.

Mean changes and fold changes from baseline in testosterone and PSA levels by treatment formulation over time are shown in Figure 1.

For all studies combined, nadir testosterone of <0.35, ≥0.35 to <0.7, ≥0.7 to <1.7 and ≥1.7 nmol/L was achieved by 96% (570/592), 3.2% (19/592), 0.34% (2/592) and 0.17% (1/592) of patients, respectively (Table 3). Nadir testosterone levels did not correlate with TNM stage.

**TABLE 3:** Testosterone concentration at baseline, nadir, maximum and end of study; testosterone suppression groups; proportion of time testosterone below target suppression and time to testosterone target suppression (ITT analysis set).

Variable	Triptorelin 3.75 mg (1‐month) *n* = 301	Triptorelin 11.25 mg (3‐month) *n* = 171	Triptorelin 22.5 mg (6‐month) *n* = 120	Triptorelin all doses ITT *N* = 592
Testosterone concentration, nmol/L [ng/dl]				
Baseline				
Mean (*SD*)	11.9 (5.5) [343 (159)]	11.6 (5.2) [334 (150)]	17.8 (7.2) [513 (207)]	13.0 (6.3)
Median (min., max.)	11.4 (0.1, 42.2) [329 (3, 1216)]	10.7 (1.4, 31.3) [308 (40, 902)]	16.8 (3.7, 49.5) [484 (107, 1427)]	12.1 (0.1, 49.5)
Nadir				
Mean (*SD*)	0.1 (0.1) [3 (3)]	0.1 (0.1) [3 (3)]	0.2 (0.1) [6 (3)]	0.1 (0.1)
Median (min., max.)	0.1 (0.1, 2) [3 (3, 58)]	0.1 (0.1, 0.8) [3 (3, 23)]	0.1 (0.05, 0.7) [3 (1, 20)]	0.1 (0.05, 2.0)
Maximum				
Mean (*SD*)	0.9 (2.1) [26 (61)]	0.8 (1.8) [23 (52)]	1.4 (4.1) [40 (118)]	1.0 (2.6)
Median (min., max.)	0.3 (0.1, 24.2) [9 (3, 697)]	0.3 (0.1, 15.0) [9 (3, 432)]	0.7 (0.3, 42.0) [20 (9, 1210)]	0.4 (0.1, 42.0)
End of study				
Last analysis visit	Day 253	Day 253	Day 337	NA
*n*	261	148	115	NA
Mean (*SD*)	0.3 (1.5) [9 (43)]	0.4 (1.5) [12 (43)]	0.8 (4.0) [23 (115)]	NA
Median (min., max.)	0.1 (0.1, 24.2) [3 (3, 697)]	0.1 (0.1, 15.0) [3 (3, 432)]	0.3 (0.05, 42.1) [9 (1, 1213)]	NA
Patients reaching nadir testosterone targets (testosterone suppression group), *n* (%)				
NT < 0.35 nmol/L (<10 ng/dl)	291 (97)	169 (99)	110 (92)	570 (96)
NT ≥ 0.35 to <0.7 nmol/L (≥10 to <20 ng/dl)	8 (2.7)	1 (0.58)	10 (8.3)	19 (3.2)
NT ≥ 0.7 to <1.7 nmol/L (≥20 to <50 ng/dl)	1 (0.33)	1 (0.58)	0	2 (0.34)
NT ≥ 1.7 nmol/L (≥50 ng/dl)	1 (0.33)	0	0	1 (0.17)
Proportion of time testosterone below suppression target, mean (*SD*), %				
<0.35 nmol/L (<10 ng/dl)	87 (24)	87 (21)	60 (31)	82 (27)
<0.7 nmol/L (<20 ng/dl)	95 (15)	94 (15)	92 (13)	94 (14)
<1.7 nmol/L (<50 ng/dl)	99 (6.8)	99 (6.8)	99 (3.7)	99 (6.3)
Time to (first) testosterone below suppression target in patients with target achieved, mean (*SD*), days				
<0.35 nmol/L^a^ (<10 ng/dl)	41.7 (27.8)	33.0 (14.8)	76.6 (45.8)	45.8 (33.2)
<0.7 nmol/L^b^ (<20 ng/dl)	34.4 (15.1)	31.5 (10.5)	42.1 (25.4)	35.1 (17.1)
<1.7 nmol/L^c^ (<50 ng/dl)	31.2 (8.0)	30.0 (7.4)	30.9 (15.7)	30.8 (9.9)

Abbreviations: ITT, intention to treat; max., maximum; min., minimum; MT, maximum testosterone; NT, nadir testosterone; *SD*, standard deviation.

^a^ Triptorelin 3.75 mg, *n* = 291; triptorelin 11.25 mg, *n* = 169; triptorelin 22.5 mg, *n* = 110.

^b^ Triptorelin 3.75 mg, *n* = 299; triptorelin 11.25 mg, *n* = 170; triptorelin 22.5 mg, *n* = 120.

^c^ Triptorelin 3.75 mg, *n* = 300; triptorelin 11.25 mg, *n* = 171; triptorelin 22.5 mg, *n* = 120.

The proportion of time that testosterone was <0.35 nmol/L ranged from 60% to 87% across the three treatments; for <0.7 nmol/L, it ranged from 92% to 95%; and for <1.7 nmol/L, it was 99% (Table 3). The time to (first) testosterone level <0.35 nmol/L ranged from 33 to 77 days across the three treatments; for <0.7 nmol/L, it ranged from 32 to 42 days; and for <1.7 nmol/L, it ranged from 30 to 32 days (Table 3). Owing to the small number of patients with nadir testosterone ≥0.7 to <1.7 nmol/L (*n* = 2) and ≥1.7 nmol/L (*n* = 1), data are not reported for these groups.

### 3.2 Overall survival

**FIGURE 2:**
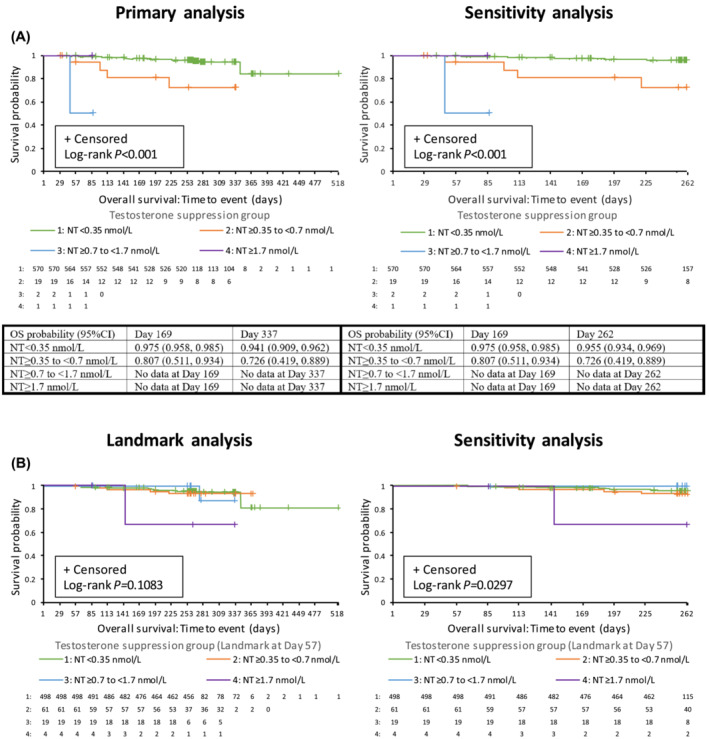
Kaplan–Meier plots of OS by testosterone suppression group for (A) primary analysis and (B) landmark analysis, with landmark at Day 57. Number of subjects at risk and OS probability estimates at Days 169 and 337 (Days 169 and 262 for sensitivity analysis) are shown underneath each graph. Data are shown for primary analysis and landmark analysis (time frame Days 1–518; left panel) and sensitivity analysis (time frame Days 1–262; right panel), with number of subjects at risk (ITT analysis set). CI, confidence interval; ITT, intention‐to‐treat; MT, maximum testosterone; NT, nadir testosterone; OS, overall survival.

The probability of survival over time (Days 1–518) was higher in the nadir testosterone <0.35 nmol/L group than in the ≥0.35 to <0.7 nmol/L group (Figure 2A). In the ≥0.7 to <1.7 nmol/L and ≥1.7 nmol/L nadir testosterone groups, the OS trend was consistent but inconclusive due to the small number of patients and short follow‐up. This trend for better OS with decreasing levels of nadir testosterone persisted in the sensitivity analysis, which considered durations from Days 1–262 (log‐rank for difference between groups, *p* < 0.001). Results from the landmark analysis of OS (landmark at Day 57), shown in Figure 2B, support these findings.

No linear trend and no statistically significant differences in OS were observed by proportion of time with testosterone levels below the suppression target or time to (first) testosterone below the suppression target (all log‐rank *p* > 0.10).

### 3.3 Disease‐specific survival

**FIGURE 3:**
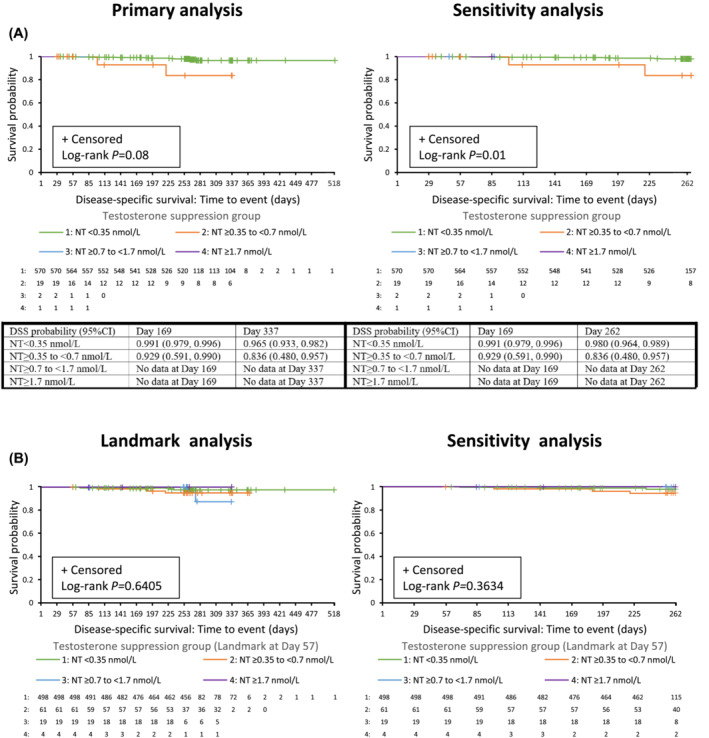
Kaplan–Meier plots of DSS by testosterone suppression group for (A) primary analysis and (B) landmark analysis, with landmark at Day 57. Number of subjects at risk and DSS probability estimates at Days 169 and 337 (Days 169 and 262 for sensitivity analysis) are shown underneath each graph. Data are shown for primary analysis and landmark analysis (time frame Days 1–518; left panel) and sensitivity analysis (time frame Days 1–262; right panel), with number of subjects at risk (intention‐to‐treat [ITT] analysis set). CI, confidence interval; DSS, disease‐specific survival; ITT, intention to treat; MT, maximum testosterone; NT, nadir testosterone.

A numerically higher probability of DSS over time was observed with decreasing levels of nadir testosterone. Specifically, for groups with adequate sample size and follow‐up, a difference in the DSS survival curves was observed between the group with nadir serum testosterone <0.35 nmol/L and the ≥0.35 to <0.7 nmol/L group (Figure 3A). However, this difference was not statistically significant (Days 1–518, log‐rank for difference between the four nadir testosterone groups *p* = 0.08). The sensitivity analysis, which considered durations from Days 1–262, supported the observed difference in DSS (log‐rank *p* < 0.05). Results from the landmark analysis of DSS (landmark at Day 57), shown in Figure 3B, support these findings.

No statistically significant differences in DSS were observed by proportion of time with testosterone levels below the suppression target or time to (first) testosterone below the suppression target (all log‐rank *p* > 0.10).

## 4 DISCUSSION

In this analysis of 592 patients with advanced prostate cancer, almost all patients (96%) achieved low nadir testosterone levels ≤0.35 nmol/L during treatment with triptorelin, and those low nadir testosterone levels correlated with improved OS. A similar nonsignificant difference was observed for DSS. Rates of OS and DSS were not affected by proportion of time with testosterone levels below the suppression target or time to (first) testosterone below the suppression target.

These findings are consistent with the pooled analysis of nine prospective studies (including the three from the current analysis), which showed that 80%–90% of patients achieved testosterone levels ≤0.7 nmol/L during 12 months of triptorelin treatment.^4^ This study shows an additional benefit with further suppression of testosterone ≤0.35 nmol/L compared with those with testosterone between 0.35 and 0.7 nmol/L. This corroborates evidence from prior studies indicating an association between lower testosterone levels during androgen‐deprivation therapy and increased OS in patients with metastatic prostate cancer.^5^ Median testosterone concentrations at the end of this study were consistent with previous observations that testosterone suppression was greater in the 1‐ and 3‐month formulations compared with the 6‐month formulation; however, variations in baseline characteristics between treatment groups may have affected the results.^4^

A testosterone nadir of ≤0.7 nmol/L correlated with improved outcomes in the PR‐7 study.^10^ A post hoc analysis of the ICELAND study found no significant differences in cause‐specific survival and time to PSA progression among testosterone level subgroups in men with advanced prostate cancer in Year 1 of androgen‐deprivation therapy.^11^ However, the ICELAND study authors noted that the results may have been due to very few patients (*n* = 3) in the highest median testosterone group. A trend in the same direction as in the PR‐7 trial was noted when patients were stratified by minimum and median testosterone levels.^11^

Although an effect of nadir testosterone group on OS was observed, the findings for DSS were less conclusive. This likely reflects, in part, the relatively few prostate cancer deaths in the cohort resulting in lack of power to demonstrate a statistically significant difference, but beyond this, the precise reasons are unclear.

A strength of the current analysis is that the included clinical studies were similar in terms of design, enrolled populations and methodology used for testosterone measurement [Correction added on 12 February 2024, after first online publication: “Limitations of this study:” has been removed from the preceding sentence.].^4^ Given the dearth of large randomised clinical trials for androgen‐deprivation therapy, this retrospective analysis helps to address a gap in the literature. Limitations include that this was a noncomparative, retrospective, single‐arm analysis. Included studies were conducted before the availability of newer targeted therapies and were too short in duration to allow for a meaningful analysis of the time to castration resistance. However, although androgen‐deprivation therapy is no longer recommended as monotherapy in current guidelines, it was at the time the trials were conducted and is still likely to be used at times in clinical practice. Most patients were treated with luteinising hormone‐releasing hormone (LHRH) agonist monotherapy, although four patients reported concomitant androgen receptor‐axis–targeted therapy and were not excluded (two patients from the DEB‐96‐TRI‐01 second phase, 3.75‐mg group and two from the DEB‐TRI6M‐3, 22.5‐mg group). Inclusion of these four patients is not expected to have a significant impact on the reported outcomes. The significance of testosterone levels in men treated with anti‐androgen or androgen receptor‐axis–targeted therapies cannot be extrapolated from these data. Testosterone suppression levels were not evaluated by age, although previous studies did not find a correlation between these variables.^5^ Nadir testosterone suppression did not appear to be associated with extent of disease; however, baseline disease stages were not balanced (most patients were T = 3, N = X and M = 1).

## 5 CONCLUSIONS

A pooled analysis of data from three Phase III studies supports the observation that very low nadir testosterone achieved during monotherapy treatment with the LHRH agonist triptorelin is associated with improved OS and DSS in patients with advanced prostate cancer. Deep testosterone suppression <0.35 nmol/L was associated with improved survival compared with those whose testosterone was between 0.35 and 0.7 nmol/L.

## AUTHOR CONTRIBUTIONS

All authors had full access to the study data and take responsibility for the integrity of the data and accuracy of the analyses. All authors significantly contributed to the interpretation of the results, the drafting and critical revision of the manuscript for important intellectual content and approved the final version.

## CONFLICT OF INTEREST STATEMENT

Laurence Klotz has received honoraria for academic presentations from Debiopharm and has also received research support from Debiopharm. Tri Tat is an employee of Debiopharm International, Lausanne, Switzerland.

## Supporting information


**Data S1.** Supporting Information.

## Data Availability

The datasets generated during and/or analysed during the current study are available from the corresponding author upon reasonable request.
